# Is there a volume-quality relationship within the independent treatment centre sector? A longitudinal analysis

**DOI:** 10.1186/s12913-019-4467-5

**Published:** 2019-11-21

**Authors:** Florien Margareth Kruse, M. C. van Nieuw Amerongen, I. Borghans, A. S. Groenewoud, E. Adang, P. P. T. Jeurissen

**Affiliations:** 1 IQ healthcare, Radboud University and Medical Center, Nijmegen, The Netherlands; 2 Dutch Health and Youth Care Inspectorate, Utrecht, The Netherlands; 3 Department of Health Evidence, Radboud University Medical Center, Nijmegen, The Netherlands; 4 Ministry of Health, Welfare and Sport, The Hague, The Netherlands

**Keywords:** Independent treatment centres, Volume, Quality of care, Outpatient procedures

## Abstract

**Background:**

The number of independent treatment centres (ITCs) has grown substantially. However, little is known as to whether the volume-quality relationship exists within this sector and whether other possible organisational factors mediate this relationship. The aim of this study is to gain a better understanding of such possible relationships.

**Methods:**

Data originate from the Dutch Health and Youth Care Inspectorate (IGJ) and the Dutch Patients Association. We used longitudinal data from 4 years (2014–2017) including three different quality measures: 1) composite of structural and process indicators, 2) postoperative infections, and 3) patient satisfaction. We measured volume by the number of invasive treatments. We adjusted for three important organisational characteristics: (1) size of workforce, (2) chain membership, and (3) ownership status. For statistical inference, random effects analysis was used. We also ran several robustness checks for the volume-quality relationship, including a fractional logit model.

**Results:**

ITCs with higher volumes scored better on structure, process and outcome (i.e. postoperative infections) indicators compared to the low-volume ITCs – although only marginally on outcome. However, ITCs with higher volumes do not have higher patient satisfaction. There is a decreasing marginal effect of volume – in other words, an L-shaped curve. The effect of the intermediating structural factors on the volume-quality relationship (i.e. workforce size, chain membership and ownership status) is less clear. Our findings suggest that chain membership has a negative influence on patient satisfaction. Furthermore, for-profit providers scored better on the Net Promoter Score.

**Conclusions:**

Our study shows with some certainty that the quality of care in low-volume ITCs is lower than in high-volume ITCs as measured by structural, process and outcome (i.e. postoperative infection) indicators. However, the size of the effect of volume on postoperative infections is small, and at higher volumes the marginal benefits (in terms of lower postoperative infections) decrease. In addition, volume is not related to patient satisfaction. Furthermore, the association between the structural intermediating factors and quality are tenuous.

**Electronic supplementary material:**

The online version of this article (10.1186/s12913-019-4467-5) contains supplementary material, which is available to authorized users.

## Background

Independent treatment centres (ITCs) are enjoying a growing market share in low-risk invasive ambulatory treatments such as cataract surgery and carpal tunnel syndrome [1–3]. The growth in ITC market share has been made possible by advances in technology, which have enabled more invasive treatments to be relocated from inpatient hospital care to ambulatory care settings [4]. In the United States (US), between 2000 and 2010, the number of Medicare-certified independent ambulatory surgery centres (referred to as ASCs in the US) increased on average by 5.4% per year [1]. In the United Kingdom (UK), the National Health Service (NHS) has increased the number of commissioned ITCs to improve accessibility and reduce waiting lists [2]. The Netherlands experienced a growth in the number of ITCs (in terms of the number of locations at which care is provided, or ‘ITC locations’), of 87% between 2009 and 2016 [5]. Although ITCs still have a small share of 3.8% of total reimbursable care in the Netherlands in 2016, for some procedures their share is considerably higher; for example, ITCs now provide 18.4% of the total ophthalmological procedures and 18.2% of the dermatological treatments [6].

The increasing importance of ITCs as providers of health care demands an understanding of the organisational factors that contribute to safe and effective care provision; however, there has been a paucity of research on this topic. Instead, most research on the ITC sector is concerned with comparing ITCs with general hospitals, and these studies often have equivocal results [7–10]. The volume-quality relationship is of particular interest in the ITC sector because organisational scale is one of the key factors in understanding efficiency.

### Dutch ITC market

The Dutch ITC market has some distinctive characteristics. It consists of non-profit centres providing reimbursable care from the statutory benefit package as well as for-profit centres offering non-reimbursable care. In the Netherlands, providers offering reimbursable medical specialist care (e.g. carpal tunnel syndrome and phlebology) from the statutory benefit package are formally prohibited from allocating any possible profits as a compensation for equity capital. Hence, stand-alone for-profit centres are clinics providing non-reimbursable care (e.g. refraction surgery and aesthetic surgery without GP referral). Many ITCs offer reimbursable and non-reimbursable care, but since they fall under the regulatory framework of reimbursable care they are strictly speaking non-profit institutions. The umbrella term ‘ITCs’ used throughout this paper refers to both non-profit and for-profit centres. Furthermore, the Dutch ITC market consists of ITC locations that are affiliated to health care chains as well as ITC locations that are sole proprietorship ITCs. The Dutch ITC market is heavily concentrated: four of the largest chains account for 32% of the total revenue [11]. Physicians working in ITCs can be working solely for an ITC but can also be partly employed by a hospital. When general physicians are working for both an ITC and a hospital, these physicians are generally on the payroll of both providers.

### Volume-quality relationship

The volume-quality relationship differs by procedure, according to the level of risk associated with it and the frequency with which hospitals undertake it (i.e. volume). Luft et al. were the first to publish on the volume-quality relationship and identified the importance of the type of procedure to the relationship [12]. Subsequently, the volume-quality relationship for high-risk, inpatient procedures has been well studied. (Although, the majority of the studies neglect the intermediating factors [13].) It has been found that lower volumes are associated with worse outcomes – often measured in postoperative mortality [14–16].

However, the contemporary debate regarding the volume-quality relationship focuses primarily on these high-risk, low-volume, inpatient procedures [17], and both low-risk, high-volume procedures and outpatient procedures have received much less attention in recent years. Some studies have examined the volume-quality relationship in low-risk, high-volume procedures but these have focused mainly on total knee and hip arthroplasty, and hernia repair surgery [15, 16, 18–22]. Moreover, almost all studies of the volume-quality relationship analyse inpatient hospital data and do not take into account care performed in outpatient settings [15–17]. Two papers by Chukmaitov et al. [23, 24] are rare exceptions, but their contribution to the evidence on the volume-quality relationship for low-risk outpatient treatments is limited because their data originates from Florida alone and is relatively outdated at the time of writing (i.e. 1997 and 2004) [23, 24].

The volume-quality relationship can move into two directions: 1) volume drives quality, and/or 2) quality drives volume [25, 26]. The first direction, wherein volume drives quality, is based on the hypothesis that ‘practice makes perfect’. This hypothesis reasons that quality is improved by harvesting experience – a learning effect which is comprised of both individual learning (i.e. experience of the surgeons) and organisational learning (i.e. skills and experience of the team and care locations) [27]. The volume-quality relationship can also be more static, meaning that high-volume providers will provide better outcomes irrespective of the experience of the provider [28]. The alternative direction of this relationship, wherein quality drives volume, is based on the hypothesis that providers that demonstrate a good quality of care will attract more patients. It is important to note that the volume-quality relationship could be characterised by either a linear or a non-linear trend [29, 30].

The theoretical framework and the empirical literature are largely focused on low-volume and high-risk treatments. (High-risk in this context does not necessarily entail a high-risk of mortality or of other severe outcomes, but it denotes negative outcomes that occur frequently.) We cannot expect that this theory can be applied directly to the ITC sector because the nature of the treatments is so fundamentally different. (The procedures are low-risk so the frequency of negative outcomes is lower than in high-risk procedures.). Hence this research adopts the null-hypothesis that there is no association between volume and quality outcomes.

### Mediating factors

To identify factors that might mediate the volume-quality association, we formulated three secondary hypotheses. The first hypothesis states that a larger workforce results in higher quality. This reflects organisational learning whereby a bigger team is associated with more internal learning, support and control, and that this then increases the quality of care. One earlier study highlighted the importance of capacity and staffing as a mediating factor in the volume-quality relationship [31].

The second hypothesis holds that chain membership leads to better quality of care. Chain-affiliated ITCs could in theory provide better quality of care, since these ITCs may enjoy the benefits of greater access to resources. The availability of complementary medical and technical support services could possibly foster broader organisational knowledge [32, 33].

The third hypothesis postulates that non-profit ITCs provide better quality of care than for-profit ITCs. Three possible explanations for this hypothesis are as follows. One theory holds that non-profit organisations will outperform for-profit entities when there is asymmetry of information in favour of the provider [34] because, according to this theory, for-profit organisations would be more inclined to game the system as a result of this asymmetry. A second theory postulates that non-profit organisations specifically strive to maximise quality, whereas for-profit ITCs aim to maximise profit for their investors [35, 36]. Furthermore, in the Netherlands, health insurers have the legal discretion to selectively contract health care providers [37]. Non-profit providers may be more incentivised to constantly improve their care because they have to compete in terms of price and quality to obtain these contracts while for-profit providers do not. However, other theories instead predict that for-profit entities outperform non-profit providers on measurable quality outputs because for-profit providers are more likely to focus on these transparent quality outputs and theoretically will outperform on them [35]. It is important to note that these theories originate from the hospital sector and we do not know in how far they hold for the ITC sector.

In summary, this study aims to explore the question of whether volume is associated with quality in the ITC sector and, in addition, identify possible mediating structural factors (i.e. workforce size, chain membership and ownership status).

## Methods

### Data

Our data originated from the Dutch Health and Youth Care Inspectorate (IGJ). IGJ uses a framework of risk indicators for the supervision of ITCs [38]. Since 2008, IGJ has been collecting annual information by means of a mandatory quality assessment questionnaire, completed by the ITC locations themselves (Additional file 1). We constructed a dataset ranging from the years 2014 to 2017. IGJ inspectors were involved in deciding which indicators were most suitable for this study. The inclusion criteria for ITCs were that they should provide invasive treatments and offer at least one of the following specialties: ophthalmology, dermatology, orthopaedics or aesthetic surgery. The dataset included 338 ITCs and 206 of these had at least 3 years of observations.

Patient satisfaction data was obtained from the Dutch Patients Association (Patientenfederatie), which collects information through a patient rating website (ZorgkaartNederland.nl). This platform is a well-known website, with around 700,000 ratings where patients, if they wish, can leave their feedback. The scores are on a 0 to 10 scale and are based on the ratings per ITC location regarding treatment, information provision, listening competency, handling by staff, accommodation, and experience in scheduling an appointment. Patient ratings between 2014 and 2017 were included. Of those ITCs included in the IGJ dataset, 166 ITCs had patient ratings. We followed the methodology of Kool et al. [39] to further restrict these scores to providers with 30 or more patient ratings, leaving 80 ITCs with a total of 19,294 ratings.

A description of how the data was merged between the patient ratings and the IGJ data can be found in Additional file 2.

### Variables

Volume was measured by the number of invasive treatments. We also constructed a percentile-based categorisation of the annual number of invasive treatments in order to gain a better understanding of how low-volume ITCs (up to ±300), lower-medium-volume ITCs (up to ±890), higher-medium-volume ITCs (up to ±2130) and high-volume ITCs perform relative to each other. (We follow the advice of Luft et al. to compare various indicators of volume [40].) Workforce size is indicated by the full-time equivalent (FTE) of physicians and nurses. Dichotomous variables were made for chain affiliation (i.e. single location versus multiple locations) and for ownership status (i.e. non-profit versus for-profit).

We used three alternative measures for different dimensions of quality: (i) a composite of structural and process indicators; (ii) postoperative infections; (iii) patient ratings. The structural and process indicators were based upon the Donabedian model [41] and are dichotomous variables with values representing ‘1’ as good performance and ‘0’ as poor performance. We constructed a composite of structural and process indicators based upon the annual sum of the Z-scores of the seven categorical structural and process quality indicators (Table 1). Z-scores were used to assign weights to the different quality measures. The data from 2017 did not have the seven categorical quality indicators, hence no observations for that year could be used for the composite measure score.

**Table 1:** The seven structural and process quality indicators

Whether an independent treatment centre…	
is reachable 24/7	
has a system whereby the performance of their personnel is reviewed	
has an arrangement in place for dysfunctional personnel	
uses a questionnaire that inquires patient reported experiences or outcomes	
classified the American Society of Anesthesiologists (ASA) physical status (i.e. severity) of their patients	
screened for delirium	
has a collaboration with (a) hospital(s)	

Medical quality was assessed by the rate of postoperative infections: the lower the rate the better the medical quality. This measure has been used for this purpose in other studies [42–44]. With the patient satisfaction data, five indicators were created: (1) promoter (average score of 9 or higher); (2) detractor (average score of 6 or lower); (3) Net Promoter Score (NPS) (i.e. the percentage of promoters minus the percentage of detractors per provider) [45]; (4) average score above 7; and (5) average score above 8. The last two measures are not based on the NPS classification but are defined to identify other possible cut-off points. Patient ratings are not normally distributed because patients who are satisfied or dissatisfied generally rate their providers more frequently than people with neutral opinions; the indicators above address this complication.

To adjust for possible confounders, four types of control variables were included in the models. Firstly, ASA physical status classification II and ASA III [46] were used to adjust for case-mix differences since this could possibly affect quality. Secondly, we adjusted for the different medical specialities since the different specialities have different quality risks. Lastly, the models account for year-dependent effects.

One of the assumptions is that locations within the same chain behave similarly. To account for chain clustering, we created unique chain identifiers.

### Data analysis

#### Descriptive statistics

Because this study uses panel data, the overall mean, the within-provider variances and between-providers variances were calculated. The differences between the overall and between variances is that the between variances use the mean of the panel data while the overall mean calculates the weighted mean of the panel data, whereby the weights are given by the number of observations in the panel data.

#### Linearity of the volume-quality relationship

For the volume-quality relationship in ITCs, linearity of the curve is tested by re-expressing the number of invasive treatments. The number of invasive treatments is right-skewed and therefore transformed down the ladder of powers – to a squared root (SQRT), a cube root (U-shaped curve) and logarithmic function (L-shaped curve) [47]. The fit of the re-expressed values is based on the Akaike information criterion (AIC) [48]. The lower the AIC score, the better the model resembles the data. To further explore this assumption, we will also report the augmented component plus residual plots according to the method proposed by Mallows [49] (consult Additional file 2 for a longer description).

#### Explanatory regressions

We used a Random Effects (RE) model which clusters the observations within the unique provider and/or chain identifiers. (The Hausman’s test preferred the RE model over the Fixed Effects estimates [50].) The continuous dependent variables (i.e. composite structural and process indicators, postoperative infections and NPS) are estimated with a linear RE model. For postoperative infections, the linear RE models only included those providers that had above 0 postoperative infections and with at least 50 invasive treatments to prevent outliers. For the binary dependent variables (i.e. promoter, detractor, average score above 7, average score above 8) a RE logistic model was used. In addition, we performed an analysis pooling all postoperative infections and invasive treatments over the 3 years to overcome the exclusion of the smaller providers with less than 50 invasive treatments and possibly include ITCs that had 0 postoperative infections in 1 year, but during the course of 3 years, are more likely to have above 0 postoperative infections. Providers with observations for only 1 or 2 years were excluded from this analysis. When providers had 4 years of observations, we took the average of the 4 years to subtract one average year from the total 4 years of observations to get 3 years of pooled observations.

The correlation between workforce size and volume can substantially distort the analysis therefore all the models were tested for multicollinearity with the variance inflation factor (VIF). We find that none of the VIF values were greater than 10 which, as a rule of thumb, suggests the models are not affected by multicollinearity [51] (Additional file 3).

For all the RE models, we tested whether observations were clustered within ITC locations and chain membership using the likelihood-ratio test. For models using longitudinal data, the test identified clustering within ITC locations. For the pooled 3-year data and the patient ratings models, the test identified clustering within chains.

#### Robustness checks

We performed a fractional logit model for postoperative infections in order to include the zeros and accommodate the proportional distribution, which the RE model is unable to do. The exclusion of the zeros could potentially penalise low-volume ITCs since they are more likely to have zero postoperative infections. The postoperative infections are included in the fractional logit model as values between 0 and 1. The fractional logit regression model can account for intragroup correlations in the panel dataset, however it is less capable than the RE model of accommodating complexities such as the unbalanced panel structure.

A second robustness check addresses the problem that within the dataset it is not possible to directly link specific treatments with specific postoperative infections because when ITCs have multiple specialties (43% of providers) total volume is assessed. To correct for this, the models with postoperative infections were also specifically run including only aesthetic surgery and postoperative infections after aesthetic surgery.

Furthermore, as a low number of invasive treatments can potentially skew the percentage of postoperative infections, an additional robustness check was performed whereby the cut-off point was set at 100 invasive treatments instead of 50. In addition, we ran the results without including the case-mix factors since many ITCs had missing values for the case-mix factors, which means the models lost a high number of providers by including case-mix as a control.

## Results

### Descriptive statistics

#### ITC characteristics

The number of invasive treatments shows substantial variation between ITCs with, on average, 1572 invasive treatments per ITC but a high standard deviation of 1882 (Table 2). With a median of 886 invasive treatments (not shown in the Table 2), this data is right-skewed. The average FTE of physicians is 2.3 physicians, with a standard deviation of 2.5 which is relatively high. Compared to the FTE of physicians, the average FTE of nurses is lower, at 1.5 nurses, with a standard deviation of 3.6, which, as for physicians, is high. Most providers are non-profit centres: 32% of the locations are for-profit. Additional file 4 summarises the differences between non-profit ITCs and for-profit ITCs with respect to volume and chain-affiliation. In brief, the non-profit ITCs are bigger than the for-profit ITCs: non-profit ITCs completed a higher number of invasive treatments. Non-profit ITCs are also more often chain-affiliated, and non-profit chains have more ITC locations than the for-profit chains. In addition, sole-proprietorship ITCs perform a lower number of invasive treatments than the chain-affiliated ITCs, and this is the case for both for-profit ITCs and non-profit ITCs.

**Table 2:** Summary statistics 2014–2017

	Overall mean ± SD	Between SD	Within SD	N (n)
Characteristics ITCs				
Number of invasive treatments	1571.85 ± 1881.56	1693.96	819.81	941 (338)
FTE physicians	2.32 ± 2.45	2.35	1.04	941 (338)
FTE nurses	1.49 ± 3.55	3.34	1.14	941 (338)
Number of locations	2.61 ± 3.11	2.73	0.82	941 (338)
Chain membership	0.40 ± 0.49	0.47	0.16	941 (338)
Non-profit providers	0.68 ± 0.47	0.47	0.00	941 (338)
Composite Quality indicators				
Reachable 24/7	0.67 ± 0.47	0.31	0.40	716 (313)
Personnel functioning system	0.78 ± 0.41	0.38	0.21	716 (313)
Personnel malfunctioning system	0.78 ± 0.41	0.36	0.22	716 (313)
Patient satisfactory questionnaire	0.88 ± 0.33	0.32	0.16	716 (313)
ASA classification known	0.48 ± 0.50	0.46	0.21	716 (313)
Screening delirium	0.34 ± 0.48	0.42	0.23	716 (313)
Collaboration with (a) hospital(s)	0.64 ± 0.48	0.45	0.20	716 (313)
Structural and process composite	−0.00 ± 3.31	3.19	1.48	716 (313)
Quality outcomes				
Percentage infections	0.28 ± 1.14	0.96	0.79	877 (318)
Percentage infections (> 0 postoperative infections & > =50 invasive treatments)	0.47 ± 0.62	0.65	0.29	412 (189)
Average patient satisfaction score	8.74 ± 1.17	0.40	1.12	19,338 (80)
Ratio promoters over total number of observations per provider	0.52 ± 0.50	0.17	0.47	19,338 (80)
Ratio detractors over total number of observations per provider	0.03 ± 0.17	0.04	0.17	19,338 (80)
Ratio 7 or more over total number of observations per provider	0.95 ± 0.22	0.04	0.21	19,338 (80)
Ratio 8 or more over total number of observations per provider	0.84 ± 0.36	0.09	0.35	19,338 (80)
Net Promoter Score (in ratio)	0.55 ± 0.19	0.19	0.06	118 (55)
Control variables				
Specialism ophthalmology	0.23 ± 0.42	0.39	0.07	941 (338)
Specialism dermatology	0.37 ± 0.48	0.47	0.13	941 (338)
Specialism orthopaedics	0.11 ± 0.31	0.31	0.05	941 (338)
Specialism aesthetic surgery	0.59 ± 0.49	0.47	0.18	941 (338)
Ratio ASA II over total number of patients	0.13 ± 0.16	0.14	0.08	622 (241)
Ratio ASA III over total number of patients	0.01 ± 0.06	0.04	0.04	623 (242)
Robustness check				
Number of aesthetic invasive treatments	502.40 ± 1269.82	972.17	844.36	488 (211)
Percentage infections after aesthetic surgery	0.90 ± 6.81	7.76	4.16	449 (182)

#### (Composite) structural and process indicator(s)

Most of the ITCs – around 70% to 80% – comply with four of the individual structural and process quality indicators, indicating that most centres perform well on these measures (Table 2). Three indicators present much lower scores of around 30 to 60%. Firstly, 36% of ITCs have no collaboration agreement with any hospital in case of emergency. Secondly, 52% of the ITCs did not use an ASA classification. And, thirdly, 66% did not screen for delirium. All of these are obligatory for ITCs conducting invasive treatments. The within standard deviation of the structural and process indicators illustrates that these indicators change over the years within ITCs. This is partly due to the fact that the weights per year could deviate. The mean of the structural and process composite is almost zero, which is as expected since the composite is based upon Z-scores. The standard deviation is 3.7, which is relatively high and demonstrates that there is substantial variance between ITCs. In order to get a sense of the scale of this composite, it ranges from − 13.1 to 5.8. (We would like to stress that this variation of Z-scores is based upon the sum of Z-scores of the seven structural and process indicators. The individual Z-scores show much less variation.)

#### Outcome indicators and patient satisfaction

The percentage of postoperative infections is low with approximately 3 in 1000 invasive treatments resulting in postoperative infections (Table 2). For those providers with at least one patient with a postoperative infection and which performed 50 or more invasive treatments, the rate was slightly higher, at 5 in 1000 invasive treatments resulting in a postoperative infection. For the outcomes related to the patient satisfaction ratings, the mean score is 8.7 with an overall standard deviation of 1.2. The mean rate of promoters lies around 52% per provider, while the mean rate of detractors accounts for 3%. The score of 7 or higher was given by 95% of the patients, and 84% score 8 or higher. The NPS accounts for 55%.

#### Control variables

There is some diversity in which specialties are offered by ITCs (Table 2). Most of the ITCs offer aesthetic surgery (59%), whereas there are fewer orthopaedic ITCs (11%). The summary statistics further show that on average 13% of the ITC patients have mild systemic diseases, ASA II, and only 1% are patients with severe systemic diseases, ASA III.

### Explanatory statistics

#### Linearity

The AIC scores of the different models are exhibited in Table 3. The relationship is non-linear for all the quality indicators; the AIC rates the logarithmic curve as the best fit for all the quality indicators.

**Table 3:** AIC scores

	Linear	SQRT	Cubic	Logarithmic
Structural and process composite	2152	2147	2144	2138
Postoperative infections	377.4	352.0	339.9	311.7
Pooled data - postoperative infections	40.90	39.10	38.60	38.30
Aesthetic postoperative infections	408.6	403.7	400.5	393.5
Patients ratings – mean score	59,435	59,433	59,432	59,430
NPS	− 128.1	−128.2	−128.3	−128.5

**Fig. 1:**
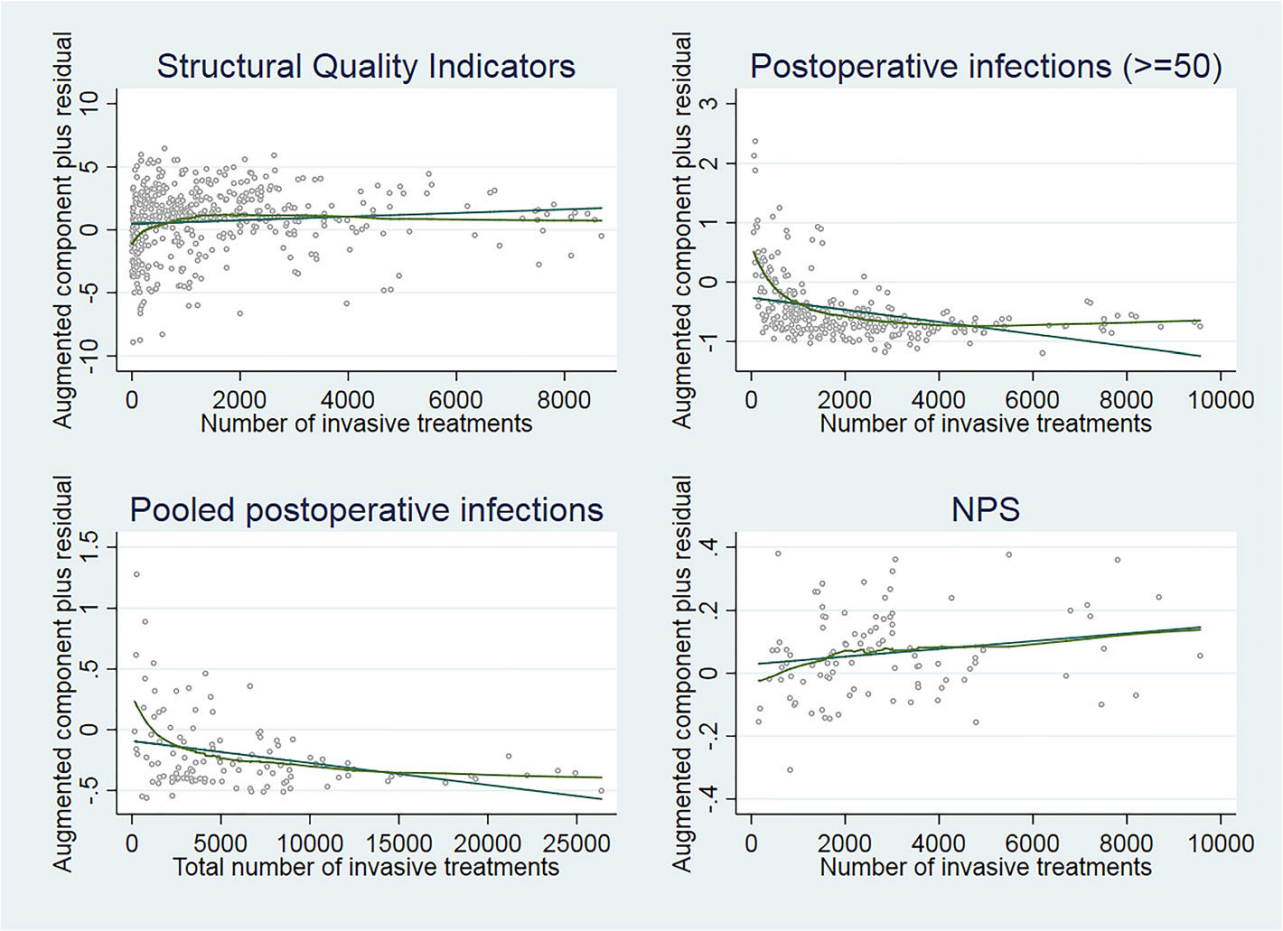
Visualisation non-linear volume-quality relationship

To visualise this relationship, Fig. 1 shows the augmented partial residuals on the y-axis and on the x-axis the total number of invasive treatments. The grey line depicts the linear trend and the green line fits to the potential non-linear curve. Unlike the AIC scores, both lines in Fig. 1 show that there is no clear non-linear trend regarding the association between volume and the structural and process indicators. Likewise, the visualisation does not present a non-linear curve for the relationship between the NPS and volume. In contrast, the observations with postoperative infections delineate a distinctive negative logarithmic function, similar to the trend found within the pooled 3-year data. For postoperative infections, the inflection point seems to occur at roughly 2000 invasive treatments; thereafter the impact of size seems to diminish.

#### Volume-quality relationship

The logarithmic curve shows a positive relationship between the composite of the structural and process indicators and volume (Table 4, model I). For ITCs with postoperative infections and with 50 or more invasive treatments, the percentage of postoperative infections declines with the number of invasive treatments (Table 4, model II). In other words, a 10% increase in the number of invasive treatments is associated with a reduction in the annual number of postoperative infections by 0.03 percentage points (− 0.339*log(1.10)). When the 3 years of observations are pooled together, the relationship persists but the effect size weakens to a 0.009 percentage point reduction in postoperative infections (− 0.094*log(1.10) (Table 4, model III). This may indicate that higher denominators and/or the exclusion of providers in the annual models (< 50 invasive treatments or 0 postoperative infections) reduce the effect size. Table 4, Model IV, suggests that low-volume ITCs have a higher chance of postoperative infections than high-volume ITCs. Patient satisfaction has a weak association with the number of invasive treatments. The mean patient rating declines with a higher number of invasive treatments (Table 5). In addition, the chance of having promoters and ratings above 8 declines with volume. All three are only statistically significant on a 90% confidence level. In contrast, the NPS, ratings above 7 and the number of detractors do not display a relationship with the number of invasive treatments.

**Table 4:** Relationship between the composite structural and process quality indicators or postoperative infections and ITC characteristics

	Model I	Model II	Model III	Model IV
*Type of outcome variable*	Composite structural and process quality indicator	Percentage postoperative infections	Percentage postoperative infections	Percentage postoperative infections
*Type of model used*	RE-Linear	RE-Linear	RE-Linear	RE-Linear
*Type of data used*	Annual data	Annual data	Total over 3 years	Annual data
Log invasive treatments	0.418*** (0.089)	−0.339*** (0.033)	−0.094*** (0.031)	
Highest quantile invasive treatments				Reference
Higher medium quantile invasive treatments				0.029 (0.096)
Lower medium quantile invasive treatments				0.169 (0.107)
Lowest quantile invasive treatments				0.293*** (0.114)
FTE number of professionals	0.009 (0.034)	0.010 (0.006)	0.011* (0.006)	0.009 (0.009)
No chain membership	Reference	Reference	Reference	Reference
Chain membership	−0.393 (0.302)	0.116* (0.063)	− 0.130 (0.090)	− 0.100 (0.074)
For-profit	Reference	Reference	Reference	Reference
Non-profit	0.449 (0.363)	0.028 (0.073)	0.174** (0.075)	0.187** (0.087)
*Cluster/Identifier*	ID ITC	ID ITC	ID Chain	ID ITC
*Observations*	459	292	112	596
*Number of groups*	211	145	72	236

Corrected for type of specialism, case-mix (i.e. ASA II & III) and year effects (except for the pooled data)

***p < 0.01, **p < 0.05, *p < 0.1

**Table 5:** Relationship between patient ratings and ITC characteristics

Type of outcome variable	Mean score	Promoter (> = 9)	Ratings > = 7	Ratings > = 8	Detractor (<=6)	NPS
Type of model	RE-Linear	RE-Logit	RE-Logit	RE-Logit	RE-Logit	RE-Linear
Log invasive treatments	−0.073* (0.035)	−0.123* (0.071)	−0.103 (0.121)	−0.174* (0.099)	0.182 (0.151)	0.027 (0.025)
FTE number of professionals	0.002 (0.003)	0.007 (0.006)	0.008 (0.012)	0.014 (0.009)	−0.013 (0.016)	−0.00 (0.002)
No chain membership	Reference	Reference	Reference	Reference	Reference	Reference
Chain membership	−0.163* (0.089)	−0.133 (0.184)	− 0.409* (0.238)	−0.390* (0.212)	0.504* (0.293)	−0.077* (0.047)
For-profit	Reference	Reference	Reference	Reference	Reference	Reference
Non-profit	−0.133 (0.114)	−0.365 (0.233)	− 0.355 (0.361)	−0.446 (0.288)	0.037 (0.429)	−0.178*** (0.061)
Level of measurement	Patient level	Patient level	Patient level	Patient level	Patient level	Provider level
Cluster	ID ITC + ID Chain	ID ITC + ID Chain	ID ITC + ID Chain	ID ITC + ID Chain	ID ITC + ID Chain	ID Chain
Observations	16,507	16,507	16,507	16,507	16,507	97
Number of groups	68	68	68	68	68	46

Corrected for case-mix (ASA II & III), type of treatment and year

***p < 0.01,  < 0.05, *p < 0.1

#### Mediating structural factors

The FTE of physicians and nurses seems to be unrelated to the structural and process quality indicators (Table 4, model I). There is no evidence of a relationship between the FTE professionals and the rate of postoperative infections in either the annual or the pooled data (Table 4, model II & III). Finally, patient satisfaction is also not significantly related to the FTE of physicians and nurses (Table 5).

Structural and process quality indicators suggest that chain membership has no effect on performance (Table 4, Model I). The positive relationship between chain membership and postoperative infections indicates that there are, on average, higher rates of postoperative infections in chain-affiliated ITCs (Table 4, model II). However, the confidence interval is only 90% and the relationship dissolves when the data is pooled (Table 4, model III). Patient satisfaction data illustrate a negative and consistent relationship with chain membership, but only on a 90% confidence interval (Table 5). The only patient satisfaction indicator which shows chain membership having no effect is the number of patients given ratings of 9 or above (i.e. promoters).

No association was found between ownership and the structural and process indicators (Table 4, model I). For the annual data analysis, non-profit providers do not seem to have a significantly higher or lower percentage of postoperative infections than for-profit providers (Table 4, model II). However, when the data is pooled, the non-profit providers are associated with higher percentages of postoperative infections (Table 4, model III). It is likely therefore that the relationship between ownership and postoperative infections can only be detected with the inclusion of higher denominators or the possible inclusion of centres that could not be included in the annual data analysis (i.e. those with < 50 invasive treatment and zero postoperative infections). Regarding the patient ratings, only the NPS is significantly lower for non-profit providers compared to for-profit providers (Table 5).

#### Robustness checks

Table 6 shows the robustness checks with fractional logit regressions and the restricted model with aesthetic invasive treatments and the percentage of postoperative infections after aesthetic surgery. The fractional logit regression results support the volume-quality findings from the RE models (Table 6, Model I & II). The only stark difference is that the lower-medium-volume ITCs also seem to perform significantly worse than the high-volume ITCs in the fractional logit regression model (Table 6, Model II). This finding possibly suggests that the inclusion of centres with zero-infections is advantageous for the relative performance of high-volume ITCs compared to the low-volume ITCs and lower-medium-volume ITCs. The restricted model with the aesthetic invasive treatments also supports the findings on the volume-quality relationship (Table 6, Model III). Lastly, the results without including the case-mix factors supports our findings in Tables 4 and 5 (Additional file 5). Interestingly, while in Table 5 there is a weak relationship between volume and patient satisfaction, without case-mix correction, all patient satisfaction indicators are negatively and significantly related to volume except for the NPS. Case-mix could partly mediate the volume-patient-satisfaction relationship, but this discrepancy can also be because the models had more statistical power due to the higher number of ITC locations included in the analysis. The model that restricted the analysis to ITCs with 100 or more invasive treatments (instead of 50 or more invasive treatments) gives similar results to the volume-quality relationship reported in Table 4 (also included in Additional file 5).

**Table 6:** Robustness check with fractional logit models and aesthetic invasive treatments

	Model I	Model II	Model III
*Type of outcome variable*	Proportional postoperative infections	Proportional postoperative infections	Percentage postoperative infections – aesthetic surgery
*Type of model used*	Fractional logit	Fractional logit	RE-Linear
*Type of data used*	Annual data	Annual data	Annual data
Log invasive treatments	−0.226** (0.111)		−0.566*** (0.135)
Highest quantile invasive treatments		Reference	
Higher medium quantile invasive treatments		0.279 (0.193)	
Lower medium quantile invasive treatments		0.869*** (0.236)	
Lowest quantile invasive treatments		1.321*** (0.452)	
FTE number of professionals	0.029 (0.021)	0.056*** (0.021)	0.012 (0.033)
No chain membership	Reference	Reference	Reference
Chain membership	−0.188 (0.215)	−0.550* (0.318)	0.755** (0.317)
For-profit	Reference	Reference	Reference
Non-profit	0.558* (0.310)	0.782** (0.339)	−0.226 (0.319)
*Cluster/Identifier*	ID ITC	ID ITC	ID ITC
*Observations*	555	596	113

Corrected for case-mix (ASA II & III), type of treatment (except Model III since it only includes aesthetic surgery) and year

****p < 0.01, **p < 0.05, *p < 0.1

## Discussion

The results of this study indicate that volume is associated with better performance on the structural and process indicators and on the number of postoperative infections – our outcome indicator. However, because the number of postoperative infections is generally low in low-risk surgical procedures, any increase in volume is associated with only a small decrease in the number of postoperative infections. Furthermore, our study suggests that there is a non-linear relationship between volume and quality, particularly for postoperative infections. This finding is in line with the findings from the hospital sector [12, 30], but contrary to the study on elective surgical procedures [18]. We find an L-shaped curve with around 2000 invasive treatments as a rough inflection point. A relationship between higher volumes and higher quality of care was also reported by Chukmaitov et al. [23], who specifically studied the ITC sector in the US and found a weak association between volume and the number of 30-day unplanned hospitalisations. The volume-quality association was also confirmed by studies scrutinising high-volume and low-risk procedures [19–22, 24, 52], and by reviews including high-risk procedures [14–16]. However, one study from the UK that looked at three elective surgical procedures (hernia repair, hip replacement and knee replacement) found no association, or of no clinical significance, between volume and quality [18].

Our models also indicate a negative relationship between volume and patient satisfaction, although with less certainty. This outcome contradicts the findings of a previous study which suggested that patients with total hip replacement surgery performed at low-volume hospitals were less satisfied than those treated in high-volume hospitals [21].

Regardless of the apparent relationship between volume and quality in this study, these findings do not provide enough evidence to reject fully the null-hypothesis because the effect size between volume and quality is small and because of limitations detailed in the limitations section. Further research should be undertaken to scrutinise the volume-quality relationship for outpatient care.

None of the three hypotheses concerning the structural mediating factors that could potentially mediate the relationship between volume and quality were supported by our study. Firstly, workforce size has no significant relationship with quality of care, and therefore our hypothesis that a bigger workforce improves quality does not hold. This outcome is contrary to one study that found a positive relationship between workforce size and quality by outpatient clinics [53]. Various studies have assessed more specifically whether surgeon volume has an effect on patient outcome. One review found a positive relationship between surgeon volume and quality of care [17]. Secondly, our study provides no evidence of a robust relationship between chain membership and quality (i.e. structure and outcome), although we did find a negative, but statistically weak (90%), association with patient satisfaction. This goes against our second hypothesis but partly reflects the evidence that shows that concentration and multihospital systems in the US hospital sector do not lead to better quality [54–57]. Thirdly, and contrary to our third hypothesis, we did not find that non-profit providers outperform for-profit providers regarding quality of care. The international empirical evidence for the relationship between ownership and quality presents mixed results which seem to depend heavily on the context (e.g. financial incentives) [58–60]. However, our findings do indicate that for-profit providers score better on the NPS – a more business-oriented, measurable outcome – which supports the theory that for-profit providers score better on the measurable and transparent outcomes.

Our findings suggest that, given the variation in quality of care among ITCs is substantial (i.e. structural and process indicators and the postoperative infections), there are various ways of improving the efficient allocation of care. On the other hand, the descriptive statistics demonstrate that on average ITCs perform well on quality. Most ITCs comply with the structural and process quality indicators; the average chance of postoperative infections is relatively low; and the average NPS is 55%, which is high compared to the median NPS of 16% for more than 400 companies in 28 industries [45].

To the best of our knowledge, this is the first scientific study on the quality of care in the ITC sector in the Netherlands and one of the first studies on the volume-quality relationship for high-volume and low-risk procedures taking the entire ITC sector into account. These findings may help various stakeholders to understand the ITC sector better. For example, the Dutch health care inspectorate inspects the ITC sector by means of inspection interventions, which in part are guided by various indicators. Some of these indicators were part of this study. The inspectorate could further investigate the difference between low- and high-volume ITCs, preferably taking into account non-linearity when using this indicator.

These results may have important implications for patients as well. In a regulated competitive health care system, patients are empowered to choose their own health care provider and our findings illustrate that patients should be aware of the variation in performance within the ITC sector.

A data-related practical implication is that the available quality indicators are sub-optimal and therefore we make an appeal to stakeholders in charge to continue their commitment to enhance quality measures within the ITC sector (e.g. patient-reported outcome measures (PROMs)) and improve the quality reporting system.

### Limitations

Despite the richness of our database there may be some biases. Firstly, we did not attempt to disentangle the direction of the volume-quality relationship. Likewise, the data did not allow us to study the learning curve of individual surgeons, with which we could have further explored the volume-quality relationship. We also did not have the opportunity to explore other mediating factors – for instance, the possible impact of quality improvement programmes [61].

Secondly, ITCs filled out the data questionnaire themselves and this could result in misreporting. It could, for example, lead to underreporting of postoperative infections due to a suboptimal postoperative surveillance system or it could incentivise desirable answers [62–64]. However, for these clinics there are no financial consequences based on what they have reported, so perverse incentives are minimised. For this reason, we expect the bias from self-reporting to move in the same direction (i.e. underreporting) for all ITCs.

Thirdly, patient ratings have their weaknesses, in particular potential selection bias [65]. A number of ITCs did not receive online patient rating scores therefore we performed a significance test with the total number ITCs included in our dataset and the ITCs with at least 30 patient ratings. The test found significant differences in relation to the size of the organisations (Additional file 6). This selection could potentially lead to a Type II error. Furthermore, the online patient rating scores might be subject to selection bias because the patients have to go proactively to the online patient rating website to provide their feedback; they do not receive a reminder after their treatment. We assume all providers are subject to the same bias.

Fourthly, it remains a challenging endeavour to assess the relationship between volume and postoperative infections because (i) the chance of having postoperative infections naturally increases with volume; (ii) small denominators can generate outliers; and (iii) the chance of having postoperative infections is rather low for ITC services. We have addressed this complexity by running a number of models: first, excluding the providers without postoperative infections and setting a minimum volume cut-off point; second, pooling 3 years of observations; and third, a fractional logit model as a robustness check.

Fifthly, although we obtained patient-level data for the patient ratings for this study, the other variables are at the ITC location level. In order to derive more conclusive results, patient-level data for all variables would be preferable, but this data does not (yet) exist for the entire ITC sector.

Lastly, we could not differentiate for hybrid locations – those ITCs that offer a combination of reimbursable and non-reimbursable care. Non-profit ITCs might avoid the for-profit ban with creative accounting [66].

## Conclusions

Our results indicate that, in general, low-volume ITCs are more likely to provide lower quality of care for low-risk invasive ambulatory care than high-volume ITCs. ITCs with more invasive treatments score better on structure, process and outcome (i.e. fewer postoperative infections). However, the relationship between volume and postoperative infections is small and is a non-linear relationship – an L-shaped curve – which suggests a ceiling whereat the marginal benefit of higher volume ITCs diminishes. The visual representation seems to suggest that the inflection point for the rate of postoperative infections is at around 2000 invasive treatments per ITC location. In addition, higher volume does not necessarily lead to higher patient ratings, and possibly even influences patient satisfaction negatively.

The mediating factors have a more tenuous relationship with quality. The size of the workforce is not related to the three quality measures. Furthermore, our results suggest that chain membership does not improve quality of care. Instead, a negative relationship between chain membership and patient ratings seems apparent. Likewise, the theory that non-profit providers outperform for-profit providers was not supported by our findings; the relationship is equivocal. Ownership type is not related to the structural and process indicators, but the findings for the pooled postoperative infections and the NPS suggest that for-profit providers might outperform non-profit providers on those quality indicators.

## Additional files

Additional file 1 Questions used from the IGJ dataset.

Additional file 2 Additional methods description.

Additional file 3 VIF scores.

Additional file 4 Summary statistics divided by the type of provider and chain-membership.

Additional file 5 Robustness checks: without ASA class included as control plus results with a cut-off point of 100 invasive treatments for postoperative infections.

Additional file 6 Statistical difference between providers with and without patients’ ratings.

## Data Availability

The longitudinal IGJ datasets (2014–2017) used and analysed during the current study are available from the corresponding author on reasonable request. The data from the Dutch Patients Association that support the findings of this study are available from the Dutch Patients Association (Patientenfederatie), but restrictions apply to the availability of these data, which were used under license for the current study, and so are not publicly available. Data are however available from the authors upon reasonable request and with permission of the Dutch Patients Association.

## Abbreviations

AIC: Akaike Information Criterion
FTE: Full time equivalent
IGJ: Dutch Health and Youth Care Inspectorate
ITC: Independent Treatment Centre
NPS: Net Promoter Score
PROMs: Patient-reported outcome measures
RE: Random Effects
SQRT: Squared root
UK: United Kingdom
US: United States
