# Supplementary material for: Is there a volume-quality relationship within the independent treatment centre sector? A longitudinal analysis
Source: BMC Health Serv Res. 2019 Nov 21;19:853. doi: 10.1186/s12913-019-4467-5 (PMC6868751; doi:10.1186/s12913-019-4467-5)
Supplement: Supplementary file 1 — Questions used from the IGJ dataset. [file 12913_2019_4467_MOESM1_ESM.docx]

**Additional file 1. Questions used from the IGJ dataset^a^**

| Aantal locaties  *Number of locations* |
| --- |
| Uw instelling is een: (ZBC, privé kliniek, medisch diagnostisch centrum)  *our location is a: (ITC, private clinic, medical diagnostic centre)* |
| Had uw instelling afspraken met een ziekenhuis waar de patiënt in geval van calamiteiten of complicaties, die niet in de particuliere kliniek of het medisch diagnostisch centrum behandelbaar zijn, terecht kan (gedurende 24 uur per dag, 7 dagen per week)?  *Did your location have an agreement with a hospital where patients, in case of complications which cannot be treated at your location, can go (24/7)?* |
| Werden refractiechirurgische ingrepen uitgevoerd in uw instelling in het verslagjaar?  *Did your location perform refractive surgery in the year of survey?* |
| Werden cataractoperaties uitgevoerd in uw instelling in het verslagjaar?  *Did your location perform cataract surgery in the year of survey?* |
| Werden orthopedische ingrepen uitgevoerd in uw instelling in het verslagjaar?  *Did your location perform orthopaedic surgery in the year of survey?* |
| Werden plastisch chirurgische ingrepen uitgevoerd in uw instelling in het verslagjaar?  *Did your location perform plastic surgery in the year of survey?* |
| Werden cosmetische ingrepen uitgevoerd in uw instelling in het verslagjaar?  *Did your location perform cosmetic surgery in the year of survey?* |
| Werden dermatologische ingrepen uitgevoerd in uw instelling in het verslagjaar?  *Did your location perform dermatological surgery in the year of survey?* |
| Voerde uw instelling in het verslagjaar invasieve ingrepen uit?  *Did your location perform invasive surgery in the year of survey?* |
| Totaal aantal invasieve behandelingen in het verslagjaar  *Total number of invasive treatments* *in the year of survey* |
| Totaal aantal patiënten dat een invasieve ingreep onderging in het verslagjaar  *Total number of patients with invasive treatments in the year of survey* |
| Kunt u onderscheid maken naar ASA-klasse?  *Can you distinguish between ASA status?* |
| Wat was het aantal patiënten in ASA-klasse 1?  *What was the number of patients with ASA status 1?* |
| Wat was het aantal patiënten in ASA-klasse 2?  *What was the number of patients with ASA status 2?* |
| Wat was het aantal patiënten in ASA-klasse 3 en hoger?  *What was the number of patients with ASA status 3 or higher?* |
| Wat was het aantal patiënten met een onbekende ASA-klasse?  *What was the number of patients with unknown ASA status?* |
| Beschikte u in het verslagjaar over een deliriumprotocol dat voldoet aan bovenstaande beschrijving?  *Did your location have a delirium protocol in the year of survey?* |
| Screende u in het verslagjaar structureel patiënten op risico voor delirium?  *Did your location screen patients on delirium a structural basis ?* |
| Aantal patiënten met een postoperatieve infectie in het verslagjaar  *Number of patients with postoperative infections in the year of survey* |
| Aantal BIG-geregistreerde basisartsen (in FTE), werkzaam in uw instelling in het verslagjaar. *Number of registered physicians (in FTE) working at your location in year of survey* |
| Aantal RGS-geregistreerde medisch-specialisten (in FTE), werkzaam in uw instelling in het verslagjaar  *Number of registered medical specialists physicians (in FTE) working at your location in year of survey* |
| Aantal (RGS-geregistreerde) medisch specialisten in het verslagjaar (NIET in FTE)  *Number of registered medical specialists physicians (not in FTE) working at your location in year of survey* |
| Aantal (BIG-geregistreerde) basisartsen in het verslagjaar (NIET in FTE)  *Number of registered physicians (not in FTE) working at your location in year of survey* |
| De behandelend arts was in het verslagjaar 24 uur per dag oproepbaar  *The physician who carried out the treatment could be reached 24/7* |
| De dienstdoende arts was in het verslagjaar 24 uur per dag oproepbaar  *The physician on duty could be reached 24/7* |
| Voerde u in het verslagjaar patiënttevredenheidsonderzoek uit?  *Did your location carry out a patient satisfactory survey?* |
| a. Note: the ITC locations fill out the questionnaires themselves. Sometimes the head office of the chain fills out the forms for all their locations, but these questionnaire are still answered by each care location separately. |
