# Supplementary material for: Is there a volume-quality relationship within the independent treatment centre sector? A longitudinal analysis
Source: BMC Health Serv Res. 2019 Nov 21;19:853. doi: 10.1186/s12913-019-4467-5 (PMC6868751; doi:10.1186/s12913-019-4467-5)
Supplement: Supplementary file 2 — Additional methods description. [file 12913_2019_4467_MOESM2_ESM.docx]

**Additional file 2. Additional methods description**

*Merge patients’ ratings and IGJ data*

There were some patients’ rating locations that did not have data with the corresponding year in the IGJ data. For those observations that could not be matched, the organisational characteristics of the ITCs were assigned to those patients’ rating observations whereby the gap of the last year’s observation in the IGJ dataset was the smallest, gaps above 3 years were excluded (86 patients’ rating observations were deleted due to this restriction).

*Augmented component plus residual plots*

The augmented component plus residual plots is a method proposed by Mallows [1] to visually detect non-linearity. The partial residuals are the dependent variables corrected for all the independent variables except the variable in question -in our case invasive treatments- and the augmented partial residuals adds a quadratic term. “In the absence of nonlinearity, the augmented partial residual plot and the component-plus-residual plot are similar. But if a nonlinear effect is present in a variable (it need not be quadratic), the augmented residual plot gives a clearer picture of the effect than the component residual plot”: [2]^p.58^. The graphs are supported by a linear line and a line that locally weights the regression, to illustrate potential non-linearity for each quality indicator with the number of invasive treatments.

**References**

1. Mallows CL: **Augmented Partial Residuals**. *Technometrics* 1986, **28**(4):313-319.<https://doi.org/10.1080/00401706.1986.10488149>

2. Chatterjee S, Hadi AS: **Sensitivity analysis in linear regression**, vol. 327: John Wiley & Sons; 2009.
