# Supplementary material for: Is there a volume-quality relationship within the independent treatment centre sector? A longitudinal analysis
Source: BMC Health Serv Res. 2019 Nov 21;19:853. doi: 10.1186/s12913-019-4467-5 (PMC6868751; doi:10.1186/s12913-019-4467-5)
Supplement: Supplementary file 3 — VIF scores. [file 12913_2019_4467_MOESM3_ESM.docx]

**Additional file 3.**

VIF scores: testing for multicollinearity

|  | Composite Quality indicator | | Postoperative infections | | 3 year pooled postoperative infections | | Aesthetic postoperative infections | | Patients ratings | | NPS | |
| --- | --- | --- | --- | --- | --- | --- | --- | --- | --- | --- | --- | --- |
|  | VIF | 1/VIF | VIF | 1/VIF | VIF | 1/VIF | VIF | 1/VIF | VIF | 1/VIF | VIF | 1/VIF |
| Log invasive treatments | 1.45 | 0.69 | 1.66 | 0.60 | 1.89 | 0.53 | 1.48 | 0.68 | 3.09 | 0.32 | 2.02 | 0.49 |
| Chain membership | 1.06 | 0.95 | 1.29 | 0.78 | 1.27 | 0.79 | 1.23 | 0.81 | 2.20 | 0.46 | 1.54 | 0.65 |
| FTE of professionals (physicians and nurses) | 1.77 | 0.56 | 2.77 | 0.36 | 2.54 | 0.39 | 1.36 | 0.73 | 7.95 | 0.13 | 3.12 | 0.32 |
| Ownership | 1.30 | 0.77 | 1.23 | 0.81 | 1.34 | 0.75 | 1.48 | 0.67 | 1.26 | 0.79 | 1.36 | 0.74 |
| Specialism ophthalmology | 1.64 | 0.61 | 1.75 | 0.57 | 2.38 | 0.42 |  |  | 3.80 | 0.26 | 3.58 | 0.28 |
| Specialism orthopedics | 1.79 | 0.56 | 2.26 | 0.44 | 2.17 | 0.46 |  |  | 5.24 | 0.19 | 2.96 | 0.34 |
| Specialism aesthetic surgery | 1.33 | 0.75 | 1.38 | 0.72 | 1.51 | 0.66 |  |  | 2.06 | 0.49 | 1.58 | 0.63 |
| Specialism dermatology | 1.47 | 0.68 | 1.42 | 0.70 | 1.77 | 0.57 |  |  | 2.57 | 0.39 | 2.84 | 0.35 |
| ASA II | 1.26 | 0.80 | 1.17 | 0.85 | 1.84 | 0.54 | 1.27 | 0.79 | 2.58 | 0.39 | 1.93 | 0.52 |
| ASA III | 1.19 | 0.84 | 1.29 | 0.77 | 1.50 | 0.67 | 1.29 | 0.78 | 3.87 | 0.26 | 2.60 | 0.38 |
| Year 2 | 1.42 | 0.71 | 1.56 | 0.64 |  |  | 1.58 | 0.63 | 2.22 | 0.45 | 2.30 | 0.44 |
| Year 3 | 1.45 | 0.69 | 1.58 | 0.63 |  |  | 1.56 | 0.64 | 3.00 | 0.33 | 3.23 | 0.31 |
| Year 4 |  |  | 1.72 | 0.58 |  |  | 1.55 | 0.64 | 2.31 | 0.43 | 2.65 | 0.38 |
| Mean VIF | 1.43 | | 1.62 | | 1.82 | | 1.42 | | 3.24 | | 2.44 | |
