# Supplementary material for: Is there a volume-quality relationship within the independent treatment centre sector? A longitudinal analysis
Source: BMC Health Serv Res. 2019 Nov 21;19:853. doi: 10.1186/s12913-019-4467-5 (PMC6868751; doi:10.1186/s12913-019-4467-5)
Supplement: Supplementary file 4 — Summary statistics divided by the type of provider and chain-membership. [file 12913_2019_4467_MOESM4_ESM.docx]

**Additional file 4.**

Summary statistics divided by the type of provider and chain-membership (2014-2017)

|  | | **Non-profit** | | **For-profit** | |
| --- | --- | --- | --- | --- | --- |
|  | | Average number of locations | Average number of invasive treatments | Average number of locations | Average number of invasive treatments |
| Chain affiliated ITC locations | Overall mean ± SD | 5.41 ± 4.08 | 2204.40 ± 2206.17 | 4.14 ± 2.52 | 1296.36 ± 2513.29 |
|  | n% | 42% | | 39% | |
| Sole proprietorship | Overall mean ± SD |  | 1495.43 ± 1647.30 |  | 988.38 ± 1020.13 |
|  | n% | 58% | | 61% | |
