# Supplementary material for: Is there a volume-quality relationship within the independent treatment centre sector? A longitudinal analysis
Source: BMC Health Serv Res. 2019 Nov 21;19:853. doi: 10.1186/s12913-019-4467-5 (PMC6868751; doi:10.1186/s12913-019-4467-5)
Supplement: Supplementary file 5 — Robustness checks: without ASA class included as control plus results with a cut-off point of 100 invasive treatments for postoperative infections. [file 12913_2019_4467_MOESM5_ESM.docx]

**Additional file 5. Robustness checks**

Results without ASA classification included as control

| Type of outcome variable | **Composite Quality indicator** | **Postoperative infections** | **Postoperative infections** | **Mean**  **score** | **Promoter (>=9)** | **Ratings >=7** | **Ratings >=8** | **Detractor (<=6)** | **NPS** |
| --- | --- | --- | --- | --- | --- | --- | --- | --- | --- |
| *Type of model* | *RE - Linear* | *RE –Linear* | *RE – Linear* | *RE – Linear* | *RME- Logit* | *RME- Logit* | *RME- Logit* | *RME- Logit* | *RE -MLE* |
| *Type of data used* | *Annual data* | *Annual data* | *Pooled over 3 years* | *Annual data* | *Annual data* | *Annual data* | *Annual data* | *Annual data* | *Pooled to provider level* |
| Log invasive treatments | 0.420***  (0.083) | -0.396***  (0.034) | -0.096***  (0.029) | -0.068**  (0.028) | -0.136**  (0.057) | 0.244**  (0.120) | -0.190**  (0.096) | -0.145**  (0.073) | 0.004  (0.021) |
| FTE number of professionals | -0.023  (0.032) | 0.017**  (0.007) | 0.008  (0.006) | 0.003  (0.003) | 0.005  (0.006) | -0.020  (0.015) | 0.014  (0.012) | 0.015*  (0.008) | -0.000  (0.002) |
| No chain membership | Reference | Reference | Reference | Reference | Reference | Reference | Reference | Reference | Reference |
| Chain membership | -0.254  (0.287) | 0.067  (0.063) | -0.108  (0.078) | -0.133*  (0.070) | -0.131  (0.143) | 0.540**  (0.229) | -0.443**  (0.186) | -0.260  (0.168) | -0.087**  (0.042) |
| For-profit | Reference | Reference | Reference | Reference | Reference | Reference | Reference | Reference | Reference |
| Non-profit | 1.402***  (0.378) | 0.144*  (0.081) | 0.140**  (0.064) | -0.144  (0.104) | -0.362  (0.236) | 0.070  (0.377) | -0.318  (0.318) | -0.560**  (0.268) | -0.164***  (0.056) |
| *Cluster/Identifier* | *ID ITC* | *ID ITC* | *ID Chain* | *ID ITC + ID Chain* | *ID ITC + ID Chain* | *ID ITC + ID Chain* | *ID ITC + ID Chain* | *ID ITC + ID Chain* | *ID Chain* |
| *Observations* | 716 | 410 | 136 | 19,294 | 19,294 | 19,294 | 19,294 | 19,294 | 117 |
| *Number of groups* | 313 | 187 | 93 | 80 | 80 | 80 | 80 | 80 | 55 |
| Controlled for type of specialism and year effects (expect for the pooled data)  *** p<0.01, ** p<0.05, * p<0.1 | | | | | | | | | |

Results with a cut-off point of 100 invasive treatments for postoperative infections

| *Type of outcome variable* | **Postoperative infections** |
| --- | --- |
| *Type of model* | RE |
| *Type of data used* | Annual data |
| Log invasive treatments | -0.270***  (0.031) |
| FTE number of professionals | 0.009*  (0.006) |
| No chain membership | Reference |
| Chain membership | 0.101*  (0.057) |
| For-profit | Reference |
| Non-profit | 0.029  (0.065) |
| *Cluster/Identifier* | ID ITC |
| *Observations* | 287 |
| *Number of groups* | 143 |
| Corrected for case-mix(ASA 2 & 3), type of treatment and year  *** p<0.01, ** p<0.05, * p<0.1 | |

‘
