# Supplementary material for: Is there a volume-quality relationship within the independent treatment centre sector? A longitudinal analysis
Source: BMC Health Serv Res. 2019 Nov 21;19:853. doi: 10.1186/s12913-019-4467-5 (PMC6868751; doi:10.1186/s12913-019-4467-5)
Supplement: Supplementary file 6 — Statistical difference between providers with and without patients’ ratings. [file 12913_2019_4467_MOESM6_ESM.docx]

**Additional file 6.**

Statistical difference between providers with and without patients’ ratings

|  | **Difference** | **T-statistics** |
| --- | --- | --- |
| Number of invasive treatments | 1368.10*** | (5.68) |
| FTE of professionals (physicians and nurses) | 4.44*** | (5.57) |
| Chain membership | -0.02 | (-0.38) |
| Ownership | 0.23*** | (4.55) |
| Specialism ophthalmology | 0.15** | (2.64) |
| Specialism orthopaedics | 0.12* | (2.61) |
| Specialism aesthetic surgery | -0.08 | (-1.27) |
| Specialism dermatology | 0.04 | (0.65) |
| ASA II | 0.07* | (2.22) |
| ASA III | 0.02** | (3.12) |
| N | 328 |  |
| * p<0.05, **p<0.01, *** p<0.001 | | |
